# Low anterior resection syndrome: An unavoidable price to pay to preserve the rectum?

**DOI:** 10.3389/fonc.2022.994720

**Published:** 2022-10-14

**Authors:** Franco Marinello, Gianluca Pellino, Eloy Espín-Basany

**Affiliations:** ^1^ Colorectal Surgery Unit – Hospital Universitari Vall d’Hebron, Universitat Autònoma de Barcelona, Barcelona, Spain; ^2^ Department of Advanced Medical and Surgical Sciences, Università degli Studi della Campania “Luigi Vanvitelli”, Naples, Italy

**Keywords:** LARS – low anterior resektion syndrome, rectal cancer, complication, sacral neuromodulation, rectal surgery

## Introduction

Rectal cancer surgery has radically evolved over the past 30 years. Multimodal treatment together with consistent use of total mesorectal excision (TME) has improved the oncological outcomes (1–3) and led to increased rates of anal sphincters preservation. In turn, this has resulted in higher incidence of functional disorders, including LARS (Low Anterior Resection Syndrome), which are often difficult to manage.

The etiology of LARS is multifactorial, but the loss of the rectum is considered as the ultimate explanation. Other mechanisms which can explain LARS are a direct lesion of the anal sphincters, damage of the nerves involved in the defecation with anal-rectal inhibitory reflex annulment, decrease of the distensibility and denervation of colonic plasty, and use of preoperative radiotherapy which might reduce the elasticity of the tissues (4, 5). Also, patients who had had a diverting ileostomy are at twice-fold risk of LARS (6).

LARS includes a wide constellation of symptoms such as fecal incontinence, urgency, stool clustering and fragmentation. Many patients also complain of urinary and sexual dysfunction (7, 8). Nevertheless, the main affection of patients who suffer LARS is a high level of unproductive fecal stool incidents which can be reported to as clustering and fragmentation, fecal urgency or a mixed pattern. Dysfunction translates into impairment of patients’ daily activities that end up in “toilet-dependence” and deterioration of their quality of life, even in the long-term (9, 10). These concerns have motivated the development of a new LARS Patient-Reported Outcome Measure tool that includes symptoms and consequences (11).

## Preventing or minimizing the impact of LARS

Since LARS is multifactorial, it is almost impossible to avoid it in many patients. The authors strongly recommend having a detailed preoperative discussion of potential bowel, urinary and sexual disorders after resection, and to capture it in the respective informed consents. This is especially relevant in those cases who require an ultra-low anastomosis to ensure transit restauration, resulting in a high risk of LARS development. The authors believe that proper information to patients might tip the balance to an abdominoperineal resection.

In order to prevent the surge of functional disorders, surgeons have tried different sorts of reconstructions such as colonic j-pouch, side-to-end colorectal anastomosis, or coloplasty. However, neorectal reservoirs have only showed better outcomes regarding stool fragmentation in the first 18 months with no further benefit beyond this point (12).

In order to minimize LARS, surgeons must raise awareness before and immediately after rectal resection to identify bowel disorders and start treatment timely, since many of the symptoms are underreported. Concerning this aspect, the BOREAL program for patients following TME resection has recently been published (13). This path consists of a series of stepwise measures determined after several postoperative assessments of LARS and continence scores over a 12-month period. Patients are escalated to different treatments based on routine check-ups at 3, 6, 9 and 12 months, starting from antidiarrheal medication, and progressing to laxative bulking agents, pelvic floor physiotherapy, transanal irrigations, and sacral neuromodulation. The results showed that the majority of patients improved with the baseline treatment, with under 15% of progression from conservative measures to further actions at 12 months with an overall compliance of 72.9% (9). This study suggested that LARS might need a comprehensive and routine evaluation in order to adjust/escalate treatment. Symptoms might ameliorate throughout the first year of reconstruction (14), but the authors of the current overview consider that some of this improvement might just be patient adaptation to their new bowel movements, justifying the need for an early action.

In some cases, the introduction of total neoadjuvant therapy (TNT) with consolidation radiotherapy, associated to local excision or a “watch and wait” regime, might reduce the recommendation for rectal resection and potential LARS.

## Available treatments for LARS

As any other functional disorder, conservative management should be introduced as a first step. Even though there is little evidence that dietary modifications are effective for LARS patients, the good results of high fiber intake in reducing the odds of fecal incontinence in the general population (15), or the use of anti-diarrheal agents such as loperamide if necessary, can also apply to LARS. The authors also recommend and instruct patients – at an early stage after reconstruction – to achieve a self-management control, changing their dietary or medication habits according to their symptoms.

Pelvic floor rehabilitation is aimed to restore muscular strength and enhance pelvic floor contractions to ensure a better defecation coordination, and it has shown positive outcomes (16). Also, biofeedback and balloon rectal distension can improve (neo)rectal capacity, maximum anal resting and squeeze pressure (17). Percutaneous tibial nerve stimulation (PTNS) can reduce urgency episodes in LARS patients compared to sham stimulation (18). A recent pilot study investigating the role of acupuncture in LARS also showed positive results in a small series of patients (19) The authors believe that a comprehensive rehabilitation program, including combined pelvic floor physiotherapy, biofeedback and PTNS, can be useful in some patients affected by LARS. However, treatment adherence is key to maintain better outcomes and many of these patients who have suffered from prolonged visits to the hospital and different treatments might not adhere.

The use of transanal irrigation (TAI) has gained popularity in the non-conservative management of LARS, with very promising results. This technique provides a mechanical flush of the colon with warm water to remove feces with defecation-free days. The good results of TAI in patients with spinal cord injuries have been replicated in patients with LARS in terms of compliance, bowel function and quality of life, with significant reduction of in the number of daily movements (7 to 1) and in median LARS score (35 to 12) with a short follow-up of 6 months (20, 21). It has also been reported that early institution of TAI (after one month of colorectal reconstruction or ileostomy closure) showed better results compared with patients on supportive therapy only. However, time availability for defecation is a vital requisite for successful results after TAI, which can rise to 45 minutes per session at 3 months (22). This required evacuation time, combined with the fact that TAI may stop providing benefits if suspended, represent the “Achilles heel” of the technique. At the author’s Institution, patients are strongly encouraged to try TAI if conservative treatment fails, especially those who mainly complain about clustering and fragmentation.

Sacral neuromodulation (SNM) has also been proposed to palliate symptoms associated with rectal resection after the optimal results reported in the treatment of fecal incontinence (23). According to a recent systematic review, including 114 patients, the overall success rate was 83.30% (24). Despite these promising results, the retrospective nature of the studies included in this review, the lack of control group, and the small numbers of patients included in each study, generated high heterogeneity and made it difficult to draw definitive conclusions. A randomized, cross-over, multicentric trial to assess the efficacy of SNM in major LARS (SANLARS Trial NCT03598231) has recently been terminated, and the data are currently being analyzed and could provide useful information for the use of this technique. The mechanism of action for which SNM might work in LARS is still unclear, especially because there is no (or almost none) rectum. In a recent study on colonic motility, a significant reduction in the sigmoid cyclic motor pattern has been observed in patients with LARS compared to healthy adults (25). This pattern, which may act as a brake helping to control normal bowel continence, can be initiated by SNM (26, 27). SNM is a two-stage procedure consisting of a test phase with a lead and a subsequent implantation of a generator in those patients in whom a good clinical response is observed. The authors have experienced that even though the lead implantation can be technically demanding due to the fibrosis in the sacral tissues induced by radiotherapy and surgical changes, it is safe in this population of patients. Since SNM is a surgical technique, it has been settled in a higher step in the treatment ladder of LARS (13, 28, 29). The authors would suggest, however, that since it is minimally invasive and performed under an ambulatory basis, it could be offered to patients who prefer it to TAI. However, since LARS presents with two broad patterns (fecal incontinence, clustering, or a combined syndrome), the best candidates that might benefit from SNM or TAI, have not been clearly identified yet. Besides, one must not forget that LARS might involve extra-intestinal symptoms such as urinary incontinence and sexual dysfunction. Since SNM is a multidimensional approach to pelvic disorders, it might be useful to palliate other non-intestinal symptoms in LARS, even though further research should be done in this direction.

Lastly, patients can be offered a definitive stoma. It is has been reported that around 6% may end up with a permanent ostomy due to LARS (30), even though this figure might not match patients’ real feelings and might be underestimated. Since many LARS patients had lived with an ileostomy after rectal resection, many express the desire to go back to the “non-toilet-dependent” status that stomas provide. The authors tend to wait for at least one or two years and offer a colostomy after failure of TAI and/or SNM.

## Discussion: Considerations to start focusing and future directions

Acknowledgment of LARS has risen in the last decade, but surgeons need to keep focusing on trying to prevent, ameliorate or palliate it. Besides the importance of performing an accurate and adequate technique of proctectomy and reconstruction, additional factors, e.g., avoiding the over-use preoperative radiotherapy, associated with higher risk of LARS after TME (10), are important. With new neoadjuvant chemotherapy schemes, some patients could avoid systematic preoperative radiotherapy with the potential of diminishing LARS. For example, the use of selective consolidation radiotherapy based on tumor response after induction neoadjuvant chemotherapy (PROSPECT trial – Clinicaltrials.gov ***NCT01515787***) might be beneficial in postoperative bowel function. The introduction of immunotherapy in locally advanced rectal cancer, which targets specific gene changes might also allow to use radiotherapy more selectively, thereby improving bowel function.

Also, accurate rectal anatomy definition at MRI scans can contribute to a better indication to preoperative radiotherapy (31). Since the use of preoperative radiotherapy is one of the main factors to perform a protective stoma, its selective use might reduce the rate of diverting stomas after TME. On the other hand, some patients with non-locally advanced rectal cancers might be offered an organ-sparing approach after preoperative chemoradiation. Further research might be needed to assess the role of transanal surgery platforms and techniques on subsequent faecal function after minimally invasive removal of transanal lesions (32).

The systematic high ligation of the inferior mesenteric artery (IMA) might be responsible of the onset of autonomic dysfunction which might translate in longer colonic transit below the sigmoid colon, decreased propagated colonic contractions and increased spastic minor contractions (33). Since the oncological outcomes are similar comparing low or high IMA ligation (34), some patients might benefit from low ligation, even though the real risk of developing LARS with high-ligation remains unclear and in some patients is mandatory to allow a tension-free colo-anal anastomosis.

Additional aspects to consider in terms of LARS research include optimization of body composition and general health status of patients, as well as the volume of operating surgeons and hospitals on subsequent LARS development. Obese patients have been reported to be at increased risk of complications following surgery for malignant gastrointestinal diseases (35), and morbidly obese patients have higher risk of LARS (36). A higher proportion of elderly patients with rectal cancer is being offered restorative surgical procedures, but frailty is frequent if proper assessment tools are utilized (37). It is therefore of outmost importance to adequately assess and prepare patients ahead of surgery, and to identify those who might be better suited for a colostomy, in order to further reduce the risk of LARS and subsequent complications. Lastly, consistent data have been suggested that rectal cancer treatment performed in referral centers is associated with reduced anastomotic leaks and complications (38, 39). Future studies should address if hospital and/or surgeon caseload can influence the subsequent risk of LARS.

There is increasing awareness among colorectal surgeons about functional outcomes after rectal resection, and many patients have started to seek out for help. Fortunately, there are different therapies to palliate LARS, but a comprehensive management algorithm with strong recommendations cannot be provided, due to the lack of high-quality data. Therefore, it is imperative to set patients’ expectations for any therapy and establish a shared decision making with them. The current algorithm for LARS is pyramid-shaped, ranging from conservative measures to definitive stoma. Obviously, non-invasive treatment must always be prioritized, but patients should be offered the opportunity to contribute to an interactive flow among other options such as TAI, SNM, or in those refractive cases, end-colostomy rather than having to go through all the steps. Treatment for LARS needs to be adapted to each individual case. For example, some patients with fecal incontinence predominant pattern or who do not want to spend time for TAI might be better candidates for SNM, while others with clustering would be better suited for TAI. An interesting future direction is to individualize treatments based on the assessment of concomitant urinary and sexual disfunction after rectal resection, along with bowel dysfunction.

Another field to develop is prehabilitation which could have an important role in preparing patients to colorectal reconstruction. Studies are being performed (PRELARS – Clinicaltrials.gov NCT04612569, CONTICARE – Clinicaltrials.gov NCT03876561) which will explore this field.

Regarding patients’ expectation, there is a need to highlight the lack of consensus regarding outcomes measures. LARS score is an excellent tool to screen patients with LARS, but it might not be useful to appreciate outcomes after treatment implantation. The Pre-Operative LARS (POLARS) score, a prediction model and nomogram that can estimate postoperative bowel function after restorative proctectomy has been reported to be useful to predict postoperative LARS and identify patients at higher risk and urge intensive treatment among them (40). The authors believe that symptom improvement measured by bowel diaries should be included in the assessment of patients after rectal surgery.

Functional impairment is an almost constant finding after TME surgery. An improvement in information to patients, developing evidence to better recommend therapies, and tailoring treatments is mandatory if we thrive for a patient-driven management of rectal surgery with a seal of excellence for both oncological and functional outcomes.

## Author contributions

All authors listed have made a substantial, direct, and intellectual contribution to the work and approved it for publication.

## Conflict of interest

The authors declare that the research was conducted in the absence of any commercial or financial relationships that could be construed as a potential conflict of interest.

## Publisher’s note

All claims expressed in this article are solely those of the authors and do not necessarily represent those of their affiliated organizations, or those of the publisher, the editors and the reviewers. Any product that may be evaluated in this article, or claim that may be made by its manufacturer, is not guaranteed or endorsed by the publisher.
